# Early diagnosis and successful treatment of paraneoplastic melanocytic proliferation

**DOI:** 10.1136/bjophthalmol-2014-305893

**Published:** 2015-04-23

**Authors:** Joyce C G Jansen, Joachim Van Calster, Jose S Pulido, Sarah L Miles, Richard G Vile, Tine Van Bergen, Catherine Cassiman, Leigh H Spielberg, Anita M Leys

**Affiliations:** 1 Department of Ophthalmology, University Hospitals Leuven, Leuven, Belgium; 2 Departments of Molecular Medicine and Ophthalmology, Mayo Clinic, Rochester, Minnesota, USA; 3 Department of Biochemistry and Microbiology, Marshall University, Huntington, West Virginia, USA; 4 Department of Immunology and Molecular Medicine Mayo Clinic, Rochester, Minnesota, USA; 5 Department of Neuroscience, University Hospitals Leuven, Leuven, Belgium; 6 Department of Ophthalmology Rotterdam Eye Hospital, Rotterdam, The Netherlands

**Keywords:** Retina, Imaging, Choroid, Neoplasia

## Abstract

**Background:**

Paraneoplastic melanocytic proliferation (bilateral diffuse uveal melanocytic proliferation, BDUMP) is a rare but devastating disease that causes progressive visual loss in patients who usually have an occult malignancy. Visual loss occurs as a result of paraneoplastic changes in the uveal tissue.

**Methods:**

In a masked fashion, the serum of two patients with BDUMP was evaluated for the presence of cultured melanocyte elongation and proliferation (CMEP) factor using cultured human melanocytes. We evaluated the efficacy of plasmapheresis as a treatment modality early in the disease in conjunction with radiation and chemotherapy.

**Results:**

The serum of the first case patient was investigated after plasmapheresis and did not demonstrate proliferation of cultured human melanocytes. The serum of the second case was evaluated prior to treatment with plasmapheresis and did induce this proliferation. These findings are in accordance with the diminution of CMEP factor after plasmapheresis. Treatment with plasmapheresis managed to stabilise the ocular disease progression in both patients.

**Conclusions:**

In the past, visual loss due to paraneoplastic melanocytic proliferation was considered progressive and irreversible. We treated two patients successfully with plasmapheresis and demonstrated a relation between CMEP factor in the serum of these patients and proliferation of cultured melanocytes.

## Introduction

Paraneoplastic melanocytic proliferation, also known as bilateral diffuse uveal melanocytic proliferation (BDUMP), is a rare paraneoplastic disorder in which melanocytic proliferation in the uveal tissue and subsequent destruction of the retina and retinal pigment epithelium (RPE) lead to rapidly progressive bilateral visual loss.^1–6^ The fundoscopic changes are characterised by the presence of both pigmented and amelanotic uveal melanocytic tumours, diffuse thickening of the uveal tract, exudative retinal detachments and multiple round or oval red patches at the level of the RPE with specific patterns of hyperfluorescence on fluorescein angiography.^2^ ^3^ Often, the diagnosis of the primary tumour is not made until after fundus changes and subsequent visual loss are recognised.^1–6^

Machemer first described the condition in 1966 as a bilateral diffuse malignant melanoma of the uvea in a patient with an abdominal tumour believed to be a primary pancreatic carcinoma.^7^ In this and many other early cases, eyes were removed, as the ocular lesions were believed to be malignant. In 1982, Barr *et al*^1^ reported four cases studied with histopathology and noted that the uveal tracts of both eyes were diffusely infiltrated by predominantly benign-appearing nevoid or spindle-shaped cells. The authors concluded that bilateral diffuse melanocytic uveal tumours associated with systemic malignant neoplasms constituted a newly recognised syndrome.

The link between the primary tumour and the ocular lesions was long poorly understood. Recently, Miles *et al*^8^ reported the detection of a specific serum-bound protein in patients with BDUMP. This protein has been found to cause in vitro melanocytic proliferation and was thus named cultured melanocyte elongation and proliferation factor (CMEP factor). Their report, along with the expanding experience in treating BDUMP and other paraneoplastic syndromes with plasmapheresis,^8–11^ prompted us to initiate therapeutic plasmapheresis immediately upon diagnosis of BDUMP, search intensively for the primary tumour and administer radiation and chemotherapy in two patients. Additionally, the serum of both patients was analysed in order to confirm the results of Miles *et al*,^8^ and determine whether the serum-induced, in vitro proliferation of cultured human melanocytes regresses following plasmapheresis.

## Methods and results

### Case 1

A 66-year-old man was referred for unexplained bilateral visual loss associated with ocular fundus changes. Extensive investigation had demonstrated neither an underlying inflammatory or infectious disease nor a lymphoma. Prior cataract surgery had not improved the visual acuity of the left eye.

Biochemical analysis of the serum, chest and abdominal CT, MRI of the brain, bone marrow aspiration, and a diagnostic vitrectomy of the left eye (OS) were all performed. Treatment with an intraocular injection of methotrexate 400 µg and a 3-day treatment course of high-dose intravenous steroids were attempted without response.

**Figure 1:**
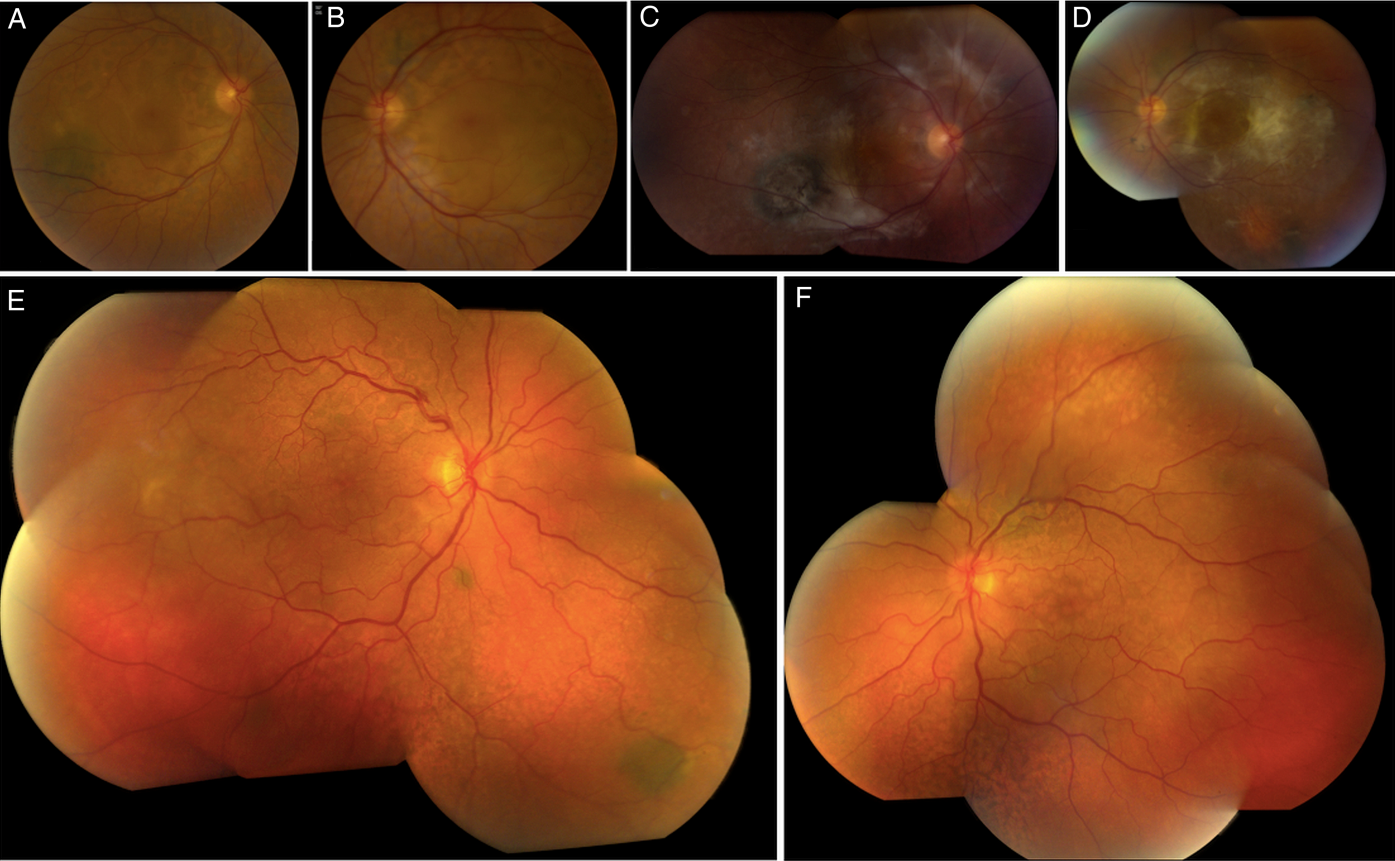
Cases 1 and 2. Clinical images of case 1 before treatment (A and B) and 29 months later (C and D). Clinical images of case 2 before treatment (E and F). Note in both eyes of both patients orange-brown patches and several nevi and note the extensive fibrotic changes post-treatment in the first patient.

**Figure 2:**
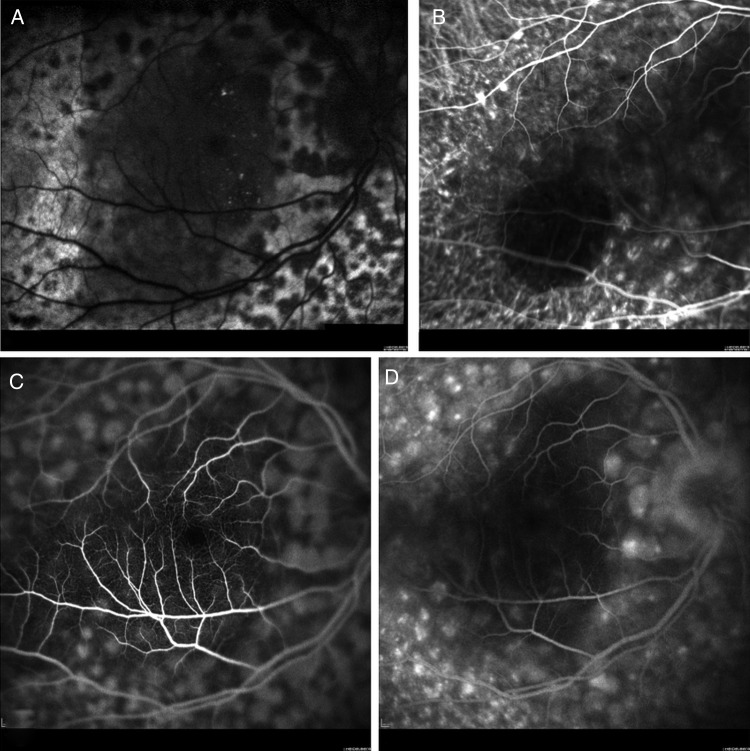
Case 1*.* (A) Autofluorescence imaging shows a characteristic mosaic of hypofluorescent spots. The central dark area is caused by serous retinal detachment masking the retinal pigment epithelium. (B) Indocyanine green angiography shows a similar mosaic with hyperfluorescent spots. The dark area in the temporal macula corresponds with a choroidal nevus. (C and D) Early and late fluorescein angiography also demonstrates the characteristic mosaic with hyperfluorescent patches and late pinpoint leakage.

**Figure 3:**
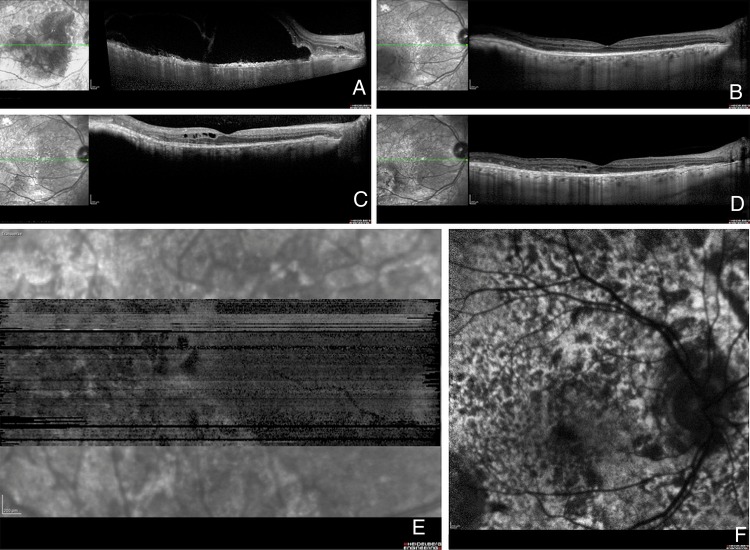
Case 1 spectral domain (SD) horizontal optical coherence tomography (OCT) scans through macula OD. (A) An enhanced-depth imaging (EDI)-OCT before treatment shows prominent pocket of subretinal fluid overlying an irregularly thickened retinal pigment epithelium (RPE). Hyperreflective spots are noted in the retina and choroid. (B) Four months after the start of treatment all fluid is resolved. (C) Cystoid retinal oedema is demonstrated 8 months after the start of treatment. (D) Inactive lesions 29 months after the start of treatment with residual cysts in the inner retina and destructive changes in the outer retina and at the level of the RPE. (E) En face OCT demonstrates a mosaic of dark spots at the level of the RPE, which corresponds to the mosaic observed on the autofluorescence map. (F) The same mosaic of dark spots as shown on the autofluorescence map.

At presentation, the best-corrected visual acuity was 20/40 in the right eye (OD) and 20/80 OS. Fundoscopy demonstrated bilateral serous retinal detachments, numerous orange-brown patches at the level of the RPE and several choroidal nevi bilaterally (figure 1A, B). The orange-brown lesions exhibited hypoautofluorescence (figure 2A) and were hyperfluorescent on both indocyanine green angiography (figure 2B) and fluorescein angiography (figure 2C). The late images of the fluorescein angiogram demonstrated pinpoint leakage (figure 2D). Spectral domain optical coherence tomography (OCT) and enhanced-depth imaging (EDI) OCT (figure 3A) of these areas revealed pronounced changes at the level of the RPE and outer retina, with pockets of subretinal fluid overlying areas with RPE loss. Hyperreflective spots were observed in the retina and choroid. Goldmann and automated perimetry visual field testing demonstrated scotomas corresponding to the ocular fundus lesions.

A tentative diagnosis of BDUMP was made. In an attempt to treat the serous detachments, ocular treatment was first initiated. Intravitreal 1.25 mg bevacizumab (Avastin, Genentech/Hoffmann-La Roche) was administered OS, and treatment with topical and systemic carbonic anhydrase inhibitors was prescribed: acetazolamide (Diamox) 250 mg once a day and dorzolamide hydrochloride ophthalmic solution 20 mg/mL (Trusopt) two times a day. However, no benefit was noted in terms of visual acuity or fundus lesions, until plasmapheresis was initiated 9 days after the tentative diagnosis. A total of seven sessions of plasma exchange were carried out during the following several weeks. In the meantime, investigations continued in search of a primary neoplasm. Positron emission tomography (PET) imaging showed enhanced activity in subclavicular, axillary and mediastinal lymph nodes. The mediastinal lymph nodes were biopsied and histopathological analysis demonstrated malignancy compatible with metastatic adenocarcinoma of the lung (cTxcN2M0). Since the primary tumour could not be localised, and surgical treatment was of course not an option, concomitant chemotherapy and radiotherapy were started 2 weeks after the first session of plasmapheresis. The chemotherapy regimen was four cycles of the standard combination therapy for lung cancer consisting of cisplatinum, in this case associated with etoposide. Fractioned external beam radiation was applied to the chest using a linear accelerator, 66 Gy in 33 fractions.

Prior to plasmapheresis, the visual acuity had deteriorated to 20/100 OD and 20/200 OS. Eight days after the start of the plasmapheresis, the serous retinal detachments had regressed markedly in both eyes. OCT examinations 4 months later demonstrated no subretinal fluid (figure 3B). The visual acuity progressively improved to 20/40 OD and 20/64 OS despite development of fibrotic lesions in the posterior pole of both eyes. Five months after the start of treatment, mild cystoid macular oedema developed in both eyes in the absence of subretinal fluid (figure 3C). This oedema responded well to reinstitution of carbonic anhydrase inhibitors that were discontinued once the subretinal fluid had regressed. Shortly later, enlarged lymph nodes were observed outside the field of radiation, and a new tumour focus was identified (rcT1aN3M1b). This led the oncologists to believe that the tumour was cisplatin-refractory, and the regimen was switched to pemetrexed monotherapy. The tumour process regressed again. One year after the last session of plasmapheresis, the patient's serum was collected and kept at −70°C for masked evaluation for the presence of CMEP, as previously described.^8^ This postplasmapheresis serum, when brought into contact with cultured melanocytes, did not lead to melanocyte proliferation. The results were consistent with inactivity of the paraneoplastic process. Plasmapheresis had cleared the blood of the proteins that induced BDUMP and radiation and chemotherapy had resulted in an inactive tumour that no longer produced the pathogenic protein. Cataract surgery was performed in the right eye 18 months after the diagnosis of the paraneoplastic process. At present, 31 months after the diagnosis of paraneoplastic melanocytic proliferation, the patient is still undergoing treatment with pemetrexed, and has neither tumour progression nor evidence of metastasis. He maintains a relatively good quality of life. His visual acuity is stable at 20/25 OD and 20/40 OS. The fundus lesions are inactive (figures 1C, D and 3D–F).

### Case 2

**Figure 4:**
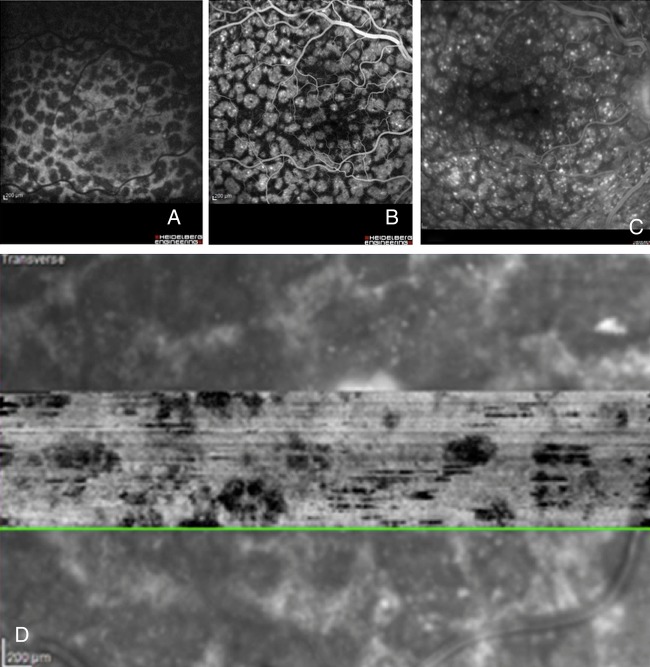
Case 2 OD. (A) Autofluorescence imaging shows characteristic hypofluorescent spots densely packed in the posterior pole. (B) The same mosaic of dark spots is observed on the optical coherence tomography en face scan positioned at the level of the retinal pigment epithelium. (C and D) The mosaic is also demonstrated on the early and late fluorescein angiogram as hyperfluorescent spots with late pinpoint leakage.

**Figure 5:**
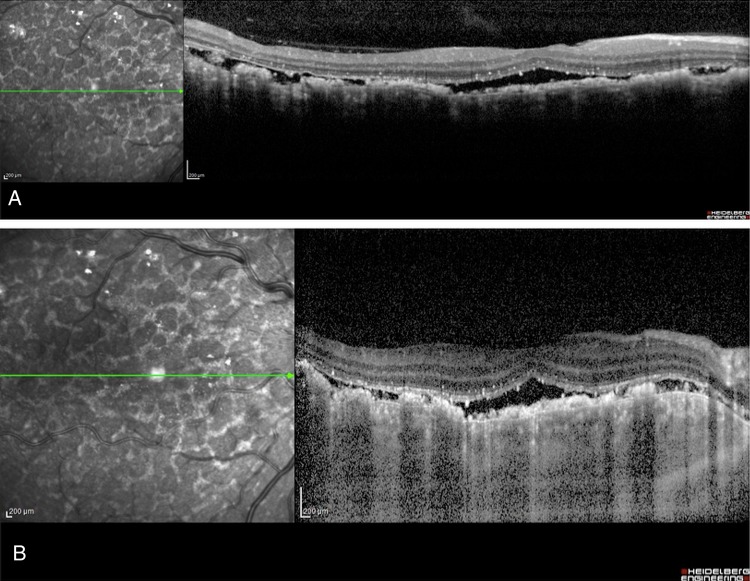
Case 2 spectral domain optical coherence tomography scan (A) and enhanced-depth imaging optical coherence tomography scan (B) of the macula OD before treatment showing alternating hyperreflective, irregularly thickened and atrophic lesions in the retinal pigment epithelium and pockets of subretinal fluid and scattered hyperreflective spots in the retina and choroid.

A 67-year-old man was referred for evaluation of rapidly progressive visual loss associated with peculiar fundus lesions bilaterally. Due to the presence of macular subretinal fluid, both eyes had been treated with one intravitreal injection of bevacizumab each shortly before referral. This had decreased the subretinal fluid and stabilised vision. Visual acuity (VA) at presentation was 20/63 both eyes. Funduscopy was suggestive for paraneoplastic melanocyte proliferation (BDUMP) with numerous bilateral dense orange lesions, several choroidal nevi and serous retinal detachments bilaterally (figure 1E, F). The orange lesions were highlighted with autofluorescence imaging (figure 4A), en face OCT (figure 4B) and fluorescein angiography (figure 4C, D), confirming the clinical suspicion of BDUMP. Extensive hypoautofluorescent lesions formed a mosaic that was also identified on en face OCT. On the fluorescein angiography, the mosaic was observed as hyperfluorescent patches associated with pinpoint leakage. Axial OCT (figure 5A) and EDI-OCT (figure 5B) demonstrated pronounced and diffuse abnormalities at the level of the RPE, with alternating hyperreflective, irregular thickening and atrophic lesions, pockets of subretinal fluid and scattered hyperreflective spots in retina and choroid. BDUMP was diagnosed and the diagnosis and treatment options were discussed with the patient. Serum was collected before systemic treatment and kept at −70°C. The frozen serum of cases 1 and 2 was evaluated at the same time by a masked investigator. A second masked investigator repeated the work, and observed the same results as the first investigator, confirming the data. The pretreatment serum of case 2 induced growth of cultured melanocytes, confirming the presence of CMEP factor and an active paraneoplastic process.

Starting 1 week after the clinical diagnosis, a total of three sessions of plasmapheresis were performed within a 1-week period, and an intensive search for a primary tumour was initiated. There was a prior history of treatment of carcinoma of the prostate, and a recent history of bowel obstruction. However, the primary tumour responsible for BDUMP proved to be a small adenocarcinoma in the right lung that had caused retroclavicular and paratracheal lymphadenopathy, which were detected on a PET scan, leading to a biopsy-proven diagnosis. Additional treatment with chemotherapy (cisplatin/taxotere) was started 5 days after the last session of plasmapheresis, followed shortly thereafter by radiotherapy, which resulted in total regression of the lung tumour and lymphadenopathy. The patient reported stabilisation of visual acuity after the start of the plasmapheresis. This subjective improvement corresponded with progressive resolution of the subretinal fluid. Five months after the start of the treatment, VA was 20/100 OD and 20/200 OS. Some measure of the vision loss was caused by cataract formation. After cataract extraction, vision improved to 20/32 OD and remained stable at 20/200 OS. At this time, the fundus lesions were inactive. Although useful vision was saved in this patient, the general prognosis for life was poor, as abdominal and intracranial metastases were detected 5 months after the diagnosis of BDUMP and the patient passed away 7 months after diagnosis.

## Discussion

BDUMP is a rare paraneoplastic disorder, with only 40 cases documented in the literature.^6^ Diagnosis of this entity is difficult, especially because a history of malignancy is often absent.^1–6^ In both cases described here, specific ocular changes were identified before the diagnosis of the primary tumour was made. Although abnormalities noted on funduscopy may be suggestive, autofluorescence, fluorescein angiography and OCT imaging contribute greatly to the diagnosis because of specific abnormalities such as alternating patches of RPE destruction and irregular thickening of RPE associated with overlying pockets of subretinal fluid and destruction of the outer retina.^2^ ^3^ ^12^ Along with knowledge of the early funduscopic features of BDUMP, high resolution imaging modalities may promote prompt diagnosis in a stage in which the patient can still benefit from treatment modalities aimed at both the paraneoplastic syndrome and the primary tumour.^9^ ^13^ Until recently, the mechanisms by which fundus changes occurred remained unclear, and visual loss as a result of BDUMP was considered irreversible.^6^ Since the report of Miles *et al*,^8^ it is clear that a primary tumour releases a pathogenic protein in the IgG fraction inducing the ocular melanocytic proliferation. Plasmapheresis can clear the serum of the BDUMP-inducing proteins. Radiation and chemotherapy can result in an inactive tumour that no longer produces the pathogenic proteins. Our first patient experienced a favourable clinical response to plasmapheresis and concomitant treatment of the underlying malignancy. The second patient had a short-term ocular benefit from plasmapheresis but passed away from an aggressive tumour with metastasis unresponsive to chemotherapy and radiation.
